# An Atomic-Level Perspective of HMG-CoA-Reductase: The Target Enzyme to Treat Hypercholesterolemia

**DOI:** 10.3390/molecules25173891

**Published:** 2020-08-26

**Authors:** Diana S. Gesto, Carlos M. S. Pereira, Nuno M. F. S. Cerqueira, Sérgio F. Sousa

**Affiliations:** 1 UCIBIO, Departamento de Química, Faculdade de Ciências e Tecnologia, Universidade Nova de Lisboa, 2829-516 Caparica, Portugal; dsgesto@fct.unl.pt; 2 UCIBIO/REQUIMTE, BioSIM, Departamento de Biomedicina, Faculdade de Medicina da Universidade do Porto, Alameda Prof. Hernâni Monteiro, 4200-319 Porto, Portugal; pereira.cmsp94@gmail.com (C.M.S.P.); nunoscerqueira@med.up.pt (N.M.F.S.C.)

**Keywords:** HMG-CoAR, structure, biochemistry, regulation, statins, dimerization inhibitors.

## Abstract

This review provides an updated atomic-level perspective regarding the enzyme 3-hydroxy-3-methylglutaryl coenzyme A reductase (HMG-CoAR), linking the more recent data on this enzyme with a structure/function interpretation. This enzyme catalyzes one of the most important steps in cholesterol biosynthesis and is regarded as one of the most important drug targets in the treatment of hypercholesterolemia. Taking this into consideration, we review in the present article several aspects of this enzyme, including its structure and biochemistry, its catalytic mechanism and different reported and proposed approaches for inhibiting this enzyme, including the commercially available statins or the possibility of using dimerization inhibitors.

## 1. Introduction

**Figure 1:**
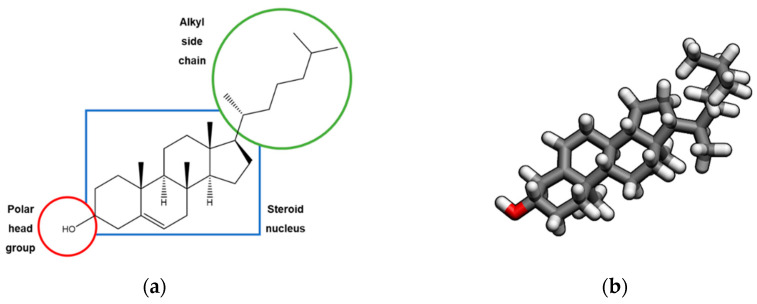
(**a**) 2D; and (**b**) 3D structures of the cholesterol molecule.

Cholesterol (Figure 1) is a molecule of vital importance to most life forms and an essential structural component of eukaryotic cells. Nevertheless, this compound is commonly associated with several heart conditions, which leads to a public perception of cholesterol as an “evil” molecule.

The chemical structure of this sterol, with the formal name cholest-5-en-3β-ol, was firstly determined in 1932 by Windaus [1]. This molecule, along with other sterols, contains a core cyclopentanoperhydrophenanthrene ring system (i.e., four fused rings, three of which are six-carbon rings and one with five carbons) and is planar, rigid and water insoluble, due its large hydrophobic hydrocarbon body. Cholesterol is also an amphiphilic molecule due to its hydrophilic head, which includes the hydroxyl group [2,3]. These properties allow this compound to be the major sterol present in animal tissues and to play a vital role in the proper functioning of our cells. Besides being an important structural lipid that can be found in the cellular membrane of most eukaryotic cells, cholesterol also works as a precursor to several steroid hormones [3,4].

Membranes, at a cellular level, are responsible for the separation of the cytosol from the external medium. They comprise very different components, although in a simplistic way they can be represented as a double layer of phospholipids. These complex systems also allow the transference of compounds through them and carry out other important functions.

The importance of cholesterol in cellular membranes can be estimated by comparing the fluidity of membranes with their cholesterol content. As cholesterol is a somewhat rigid molecule, membranes with a higher cholesterol composition tend to be more rigid and packed, and those with less tend to be more fluid [2]. The fluidity of membranes is diminished not only due to the rigidity of cholesterol, but can also be influenced by the interaction of this molecule with the different membrane lipids (e.g., the creation of cholesterol–sphingolipid rafts makes membranes slightly thicker and more ordered) [3,5]. Additionally, this membrane lipid also aids the cell in other homeostasis processes, such as endocytosis [3].

**Figure 2:**
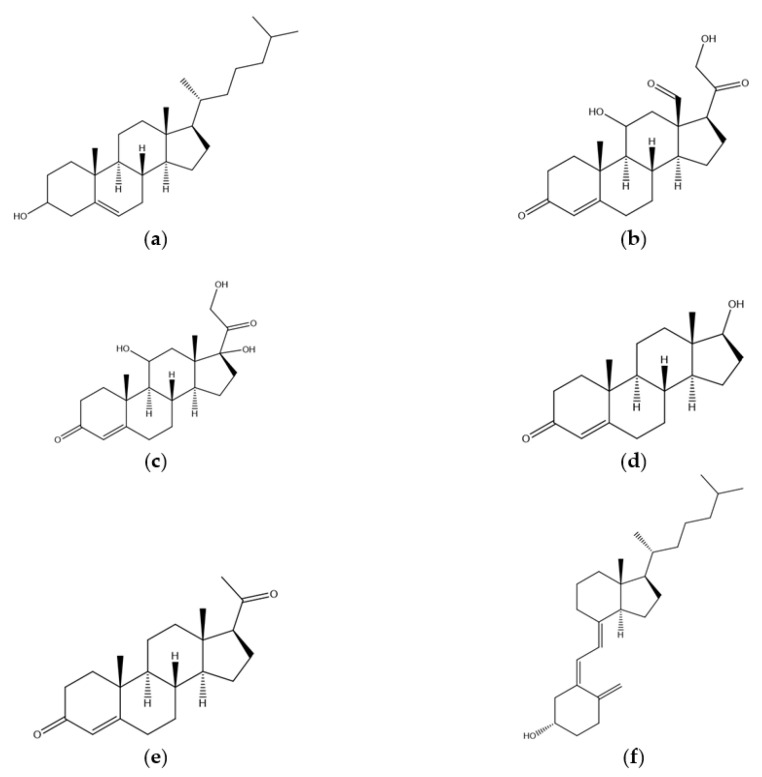
2D structures of: (**a**) cholesterol; and some of its precursors: (**b**) aldosterone; (**c**) cortisol; (**d**) testosterone; (**e**) progesterone; and (**f**) vitamin-D3.

As mentioned above, cholesterol is also a precursor of natural steroid hormones produced in our body, providing them with their core ring system, such as the hormones produced in the adrenal gland and the sex hormones (Figure 2).

The adrenal gland is located just above the kidneys and its main role is to produce and release two classes of hormones: corticosteroids and catecholamines. Only corticosteroid hormones are derived from cholesterol, and these can be further divided into two different classes: mineralocorticoids and glucocorticoids. Mineralocorticoids (e.g., aldosterone (Figure 2b)) are hormones related to the reabsorption of ions such as Cl-, Na+ and HCO3- by the kidney. On the other hand, glucocorticoids (e.g., cortisol (Figure 2c)) are a centerpiece for the metabolism of carbohydrates in the body, being responsible for the regulation of the levels of glucose. Both male and female sex glands also produce molecules that can be derived from cholesterol. These can be divided into androgens, such as testosterone (Figure 2d), which are responsible for the development of male characteristics, and estrogens and progestogens (Figure 2e), which are responsible for the development of female characteristics. Additionally, cholesterol can also be used as a precursor in the synthesis of bile salts and vitamin D (Figure 2f) [3,4].

Cholesterol present in our bodies derives from two different sources: it can be either synthesized de novo in our cells or obtained through the ingestion and absorption of certain foods, such as beef and pork meat, eggs and cheese. Although many people regularly eat these foods, there really is no absolute need to ingest them for the sole purpose of obtaining more cholesterol, since cholesterol homeostasis is regulated by the interplay between de novo synthesis/ingestion and the excretion or conversion of cholesterol into bile acids [6,7]. This means that, when low quantities of cholesterol are ingested, absorption and synthesis will be upregulated. Likewise, if the dietary intake is high, its excretion will increase [6,7]. This is a simplified approach to the cholesterol regulation mechanism, as it can change with several other external factors, e.g., aging [8]. As mentioned above, cholesterol has gained a bad reputation among the general population, especially due to its association with cardiovascular diseases (CVD). According to the World Health Association (WHO), CVDs accounted for 31% of the 57 million deaths that occurred in 2016 [9]. In the top 10 leading causes of death (worldwide) in 2016, ischemic heart disease (IHD) was number one, accounting for 16.6% of total deaths, followed by stroke and other cerebrovascular diseases (10.2%) [10]. The association between these diseases with hypercholesterolemia leads to a strong perception of cholesterol as a malignant compound by the general public.

Cholesterol is known to be associated with atherosclerosis, which is one of the main causes of CVDs [7,11]. Atherosclerosis derives from the accumulation of plaque, composed by fat, cholesterol, calcium and other substances found in the bloodstream, on the inside walls of arteries [12,13,14]. It is a complex process, which involves a chronic inflammatory response on the walls of arteries to oxidized low-density lipoproteins (LDL). This leads to a pathogenic accumulation of LDL in blood vessels and the formation of atherosclerotic plaques, which results in the constriction of blood vessels [12,13]. Lipoproteins are produced by our body in order to dilute cholesterol and other fats, which are water-insoluble, in our bloodstream. With the aid of apolipoproteins, it is possible to package these insoluble materials into protein-covered molecular assemblies. However, whenever there is a higher lipid content in these complexes, LDL (the often called “bad cholesterol”) is formed.

Even though CVDs are associated with the cholesterol content, it is important to note that high levels of cholesterol are not directly the cause of these diseases, and that people with the inability to produce this molecule have serious diseases, such as Smith–Lemli–Opitz syndrome and desmosterolosis [7,11].

## 2. Biosynthesis of Cholesterol and the Role of HMG-CoA

**Figure 3:**
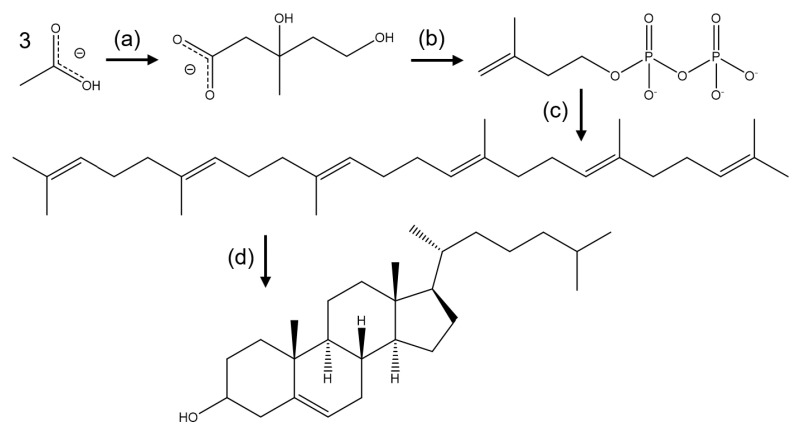
Summary of cholesterol biosynthesis. (**a**) Synthesis of mevalonate from acetate; (**b**) Conversion of mevalonate to activated isoprenes; (**c**) formation of squalene; (**d**) conversion of squalene to form the ring steroid nucleus.

Cholesterol synthesis (Figure 3) is performed by multiple cells in the human body, whenever the cholesterol content attained by ingestion is low. This complex process is normally performed in the cytoplasm, and the major contributors are the liver and intestinal tissues. The cells are, by themselves, capable of producing enough cholesterol for our needs [15,16,17]. This process is heavily regulated at several points throughout its progression, and the reaction intermediaries can be deviated to the production of other compounds and to perform other body functions [18]. This process requires several steps, which can be summarized in four stages: synthesis of mevalonate (Figure 3a); conversion of mevalonate to activated isoprenes (Figure 3b); formation of squalene (Figure 3c); and ring closure of squalene to form the sterol ring system (Figure 3d) [19,20].

**Figure 4:**
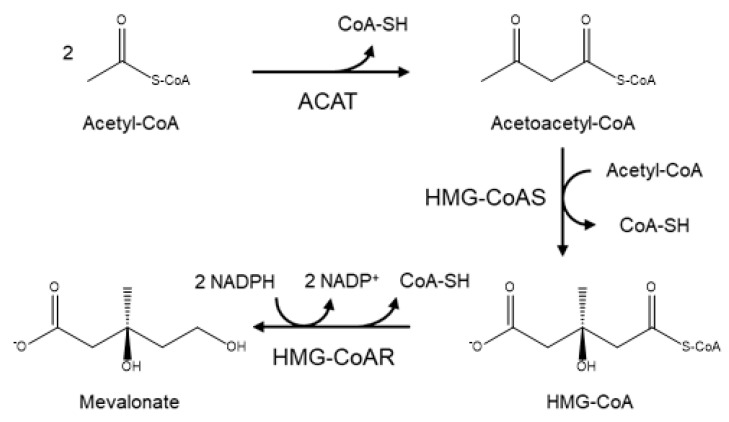
Diagram of the mevalonate pathway, the first stage in the biosynthesis of cholesterol.

In a more detailed mechanism, the synthesis of cholesterol starts with the condensation of two acetyl-coenzyme A (acetyl-CoA) molecules to form the intermediate acetoacetyl-CoA. This process is catalyzed by a thiolase enzyme, known as acetyl-CoA acetyltransferase (ACAT). Hereinafter, the reaction of two acetoacetyl-CoA molecules catalyzed by HMG-CoA synthase (HMG-CoAS) allows for the formation of 3 hydroxy-3-methylglutaryl CoA (HMG-CoA), which is subsequently reduced to mevalonate by the enzyme HMG-CoA reductase (HMG-CoAR) and two NADPH molecules that function as cofactors (Figure 4). This latter reaction is the rate-limiting step of the overall synthesis of cholesterol, and it has been extensively studied since it defines the course of the reaction, known as the committed step, using HMG-CoAR as a regulatory enzyme.

The next step in the biosynthesis of cholesterol is the conversion of the intermediate mevalonate into two activated isoprenoids—isopentenyl-5-pyrophosphate and dimethylallyl pyrophosphate (IPP)—by the addition of three ATP molecules (Figure 3b). The obtained isoprenoids are used to synthesize several biomolecules, such as cholesterol, ubiquinone and sterol-based hormones in animals; carotenoids in plants; and cell-wall components (such as undecaprenyl phosphate) in eubacteria [19,21,22,23,24,25].

A successive condensation reaction of activated isoprenes allows for the formation of a 30-carbon molecule, known as squalene (Figure 3c), whose linear structure can be linked to the cyclic steroids and act as the biochemical precursor of all steroids. The synthesis of cholesterol is achieved by the mono oxidation of squalene into an epoxide using a NADPH molecule, followed by the cyclization of the intermediate to form a 4-ring compound, the lanosterol molecule, which is converted into cholesterol after multiple subsequent reactions.

Since cholesterol biosynthesis is an energetically complex and expensive process (involves 18 ATP molecules), it is only natural to assume that it is carefully regulated. In mammals, its regulation is controlled by the intracellular cholesterol concentration and the presence of the hormones insulin and glucagon [19]. As mentioned above, the most important checkpoint is the conversion of HMG-CoA to mevalonate, a step catalyzed by HMG-CoAR. The importance of this enzyme to the mevalonate pathway has been evaluated through various experimental works, such as the work of Chappell et al., in which, after introducing a constitutively expressed HMG-CoAR gene from a hamster into tobacco plants, the activity of the enzyme became unregulated and the accumulation of sterols increased 3–10-fold [26]. The studies surrounding this enzyme were performed to assess not only its importance in the biosynthesis of cholesterol and other steroids, but also to understand how it works and is regulated.

## 3. Structure of HMG-CoA Reductase

HMG-CoAR is a very important enzyme in the production and regulation of several essential compounds. As discussed above, mevalonate will lead to the formation of isoprenoids, which are a metabolite of major importance in several different organisms, ranging from bacteria to plants and animals [21,27]. For this reason, these organisms also have similar enzymes that perform analogous reactions. HMG-CoAR can be found in eukaryotes and some prokaryotes, with some differences observed. With a sequence analysis of this protein, it was possible to point out two different classes. Class I enzymes (EC: 1.1.1.34) are present in most archaea and eukaryotes and are inserted in the membrane of the endoplasmic reticulum (ER) [27,28]. Class II HMG-CoAR (EC: 1.1.1.88), on the other hand, is completely soluble in the cytoplasm and is found in some archaea and prokaryotes [29].

**Figure 5:**
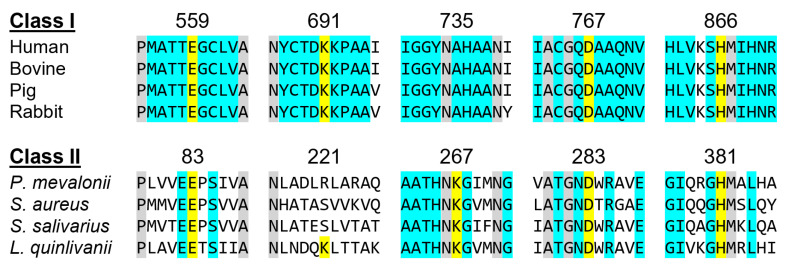
HMGCRs sequence alignment around the main catalytic residues, which are highlighted in yellow. The numbering is related to the human enzyme for Class I and with the *Pseudomonas mevalonii* one for Class II. Residues colored in grey are conserved between classes and those colored in light blue are conserved within the same class.

In a more detailed view, Class I enzymes have a transmembrane domain, ranging from residues 1 to 339; a cytosolic domain in which the active site is located (residues 460–888); and are connected by a linker region, which is comprised by residues 340–459 (the description of the residues is associated with human protein) [30]. Class II contains only the catalytic domain of the Class I enzyme, thus resulting in a 428-residue polypeptide [31,32,33]. This results in sequence identities ranging 14–20% [27,30,33]. Despite this low sequence homology between classes, as well as a different protein architecture, both enzymes have comparable positions for the active sites residues and a highly conserved catalytic portion (Figure 5) [30,33]. The study that led to the early development and understanding of the mevalonate pathway used this feature, as the first Class II HMG-CoAR enzyme was obtained from the eubacterium *Pseudomonas mevalonii* [34,35]. This enzyme was a useful model for the characterization of the active site residues, even preceding crystallographic information [34,35,36,37,38].

**Figure 6:**
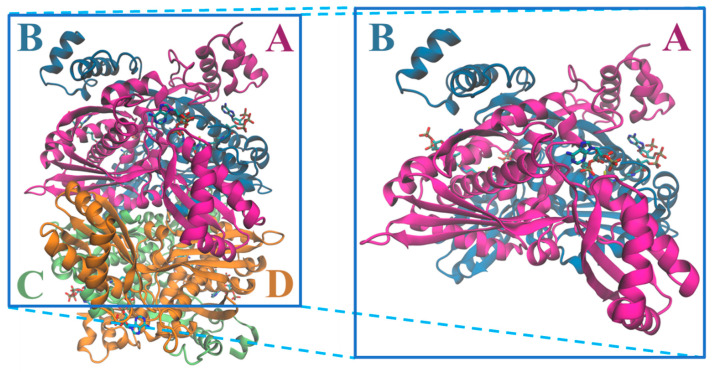
Structure of the tetramer and respective dimer of the human HMGCR, showing both CoA and NADPH (PDB entry 1DQA); chains are colored according with the letters: chain A is in magenta, chain B in blue, chain C in light green and chain D in orange.

The tridimensional structural information for the human HMGCR (Figure 6) was solved for the first time in 2000 by Istvan et al. [38]. Currently, there are 22 structures for the catalytic domain of the human enzyme available on the Protein Data Bank, which sum up to more than 40 if we also count those from other organisms. These structures contain those of the enzyme alone as well as complexes between HMGCR and HMG-CoA, NADP+ and different statins (Table 1).

**Figure 7:**
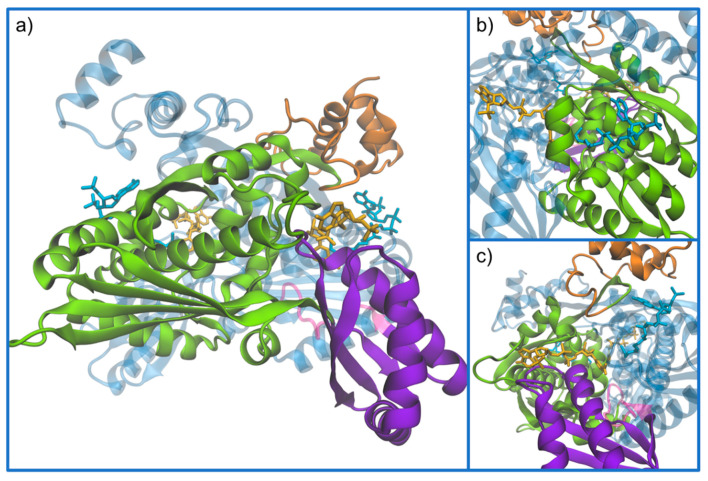
(**a**) Demonstration of the monomer subdomains. The structures follow the representation and color code of the dimer in Figure 6, as chain A and B are colored in magenta and blue (transparent to better see details), respectively. The chain A monomer is subdivided into its subdomains, highlighted in orange is the N-domain; in green the L-domain; in violet the S-domain; and the molecules represented are CoA (in light blue) and NADPH (in yellow). (**b**,**c**) Close-up representations of the CoA and NADPH-binding regions, respectively (PDB entry 1DQA).

As referred above, the Class I protein is divided in three different domains: the membrane-anchor, a linker and a catalytic domain. From the human HMGCR structures, which only include the catalytic domain, it is possible to observe a tetramer produced by four identical monomers. The monomers form dimers in which each subunit is coiled around the other in an intricate way. Each tetramer contains four active sites, two in each dimer, which are made up of residues from both subunits. The monomer itself can be divided into three different subdomains (Figure 7): A small, α-helical amino-terminal N-domain, residues 460–527; a large central L-domain that binds to HMG-CoA, residues 528–590 and 694–872 (Figure 7b); and a small carboxyl-terminal S-domain that binds to NADPH, residues 591–682 (Figure 7c) [30,33,49,50].

**Figure 8:**
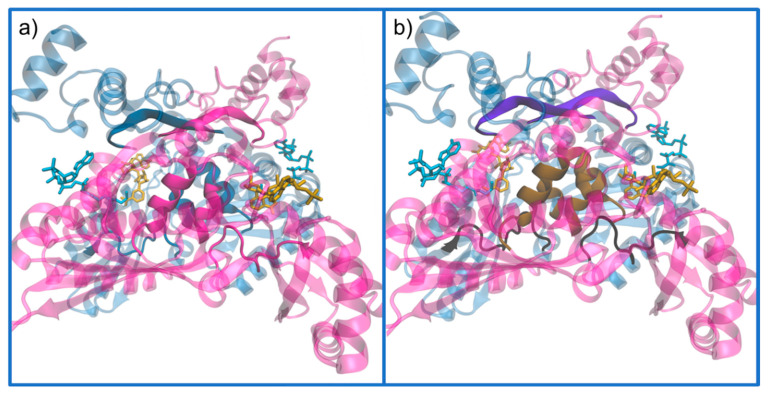
Demonstration of the dimer interfaces. These structures follow the representation and color code of the dimer in Figure 7, as chain A and B are colored in magenta and blue (transparent to better see details), respectively. (**a**) The monomer–monomer interactions are detailed by chain; and (**b**) the characterization of the regions where the interactions are stronger, where the *cis*-loop, is in black; the β-sheet in violet; and the four α-helix complex in brown (PDB entry 1DQA).

The dimer interfaces are extensive, and all domains of the monomer participate in interactions that join the subunits together. The most broad interactions are located in the three following regions (Figure 8): (i) the loop that connects the L-domain and the S-domain, residues 682–694, called the *cis*-loop, which is essential for the formation of the HMG-binding site and the connection with the NADPH-binding region; (ii) the region in the L-domain where an intramolecular β-sheet is formed, and the two strands of this β-sheet are characterized by a highly conserved sequence, ENVIGX_3_I/LP; and (iii) a four α-helix bundle formed by helices Lα6 and Lα7 (two from each monomer), which fold in an antiparallel fashion with the corresponding helices from the neighboring and equivalent subunit [49].

The core active site of this enzyme for the reduction of HMG-CoA is found in the *cis*-loop, and the key catalytic residues are Lys691, Glu559, Asp767 and His866 [30,33].

Despite the fact that the HMG-CoAR tetramer was only observed on crystallized structures of the catalytic domain alone, other studies suggest that this configuration is maintained even in the full-length human HMG-CoAR, containing both catalytic and transmembrane domains [38]. In the full-length protein, the N-domain is essential for establishing the connections between the catalytic portion of HMG-CoAR and the membrane domain [30,38].

**Figure 9:**
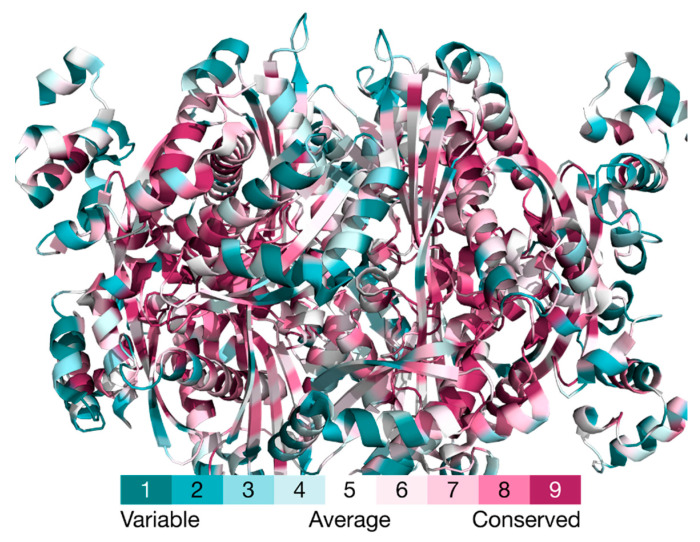
Representation of the conservation score obtained for the catalytic portion of human HMG-CoAR (PDB code 1DQA), using the software ConSurf-DB [55]. It is possible to observe in magenta highly conserved residues, typically with some structural and/or functional importance; the residues with low conservation are in cyan.

Contrary to the catalytic portion of the HMG-CoAR, the transmembrane domain is not very well conserved in eukaryotes (Figure 9). In mammals, the membrane spanning region forms eight helices that are inserted in the membrane; contrarily, plants and yeast contain two and seven, respectively [38]. Mammalian enzymes contain a 167-residue segment which is sensitive to sterols. This segment has a sequence identity of approximately 25% with other cholesterol-related membrane proteins that sense cholesterol abundance or transport cholesterol [49,51,52,53,54].

### Active Site Architecture and Catalytic Mechanism of HMG-CoA Reductase

The description of the active site of HMG-CoAR, enzyme responsible for the reduction of HMG-CoA to mevalonate, is briefly depicted above. In short, the active site of this enzyme is formed by two different subunits that form a dimer when bound together. The catalytic domain remains unchanged even with the formation of the tetramer.

The active site of the HMG-CoAR enzyme is a large cavity which is located at the monomer–monomer interface. This active site can be divided into three different binding subsites: one for HMG, one for CoA and one for NADPH.

**Figure 10:**
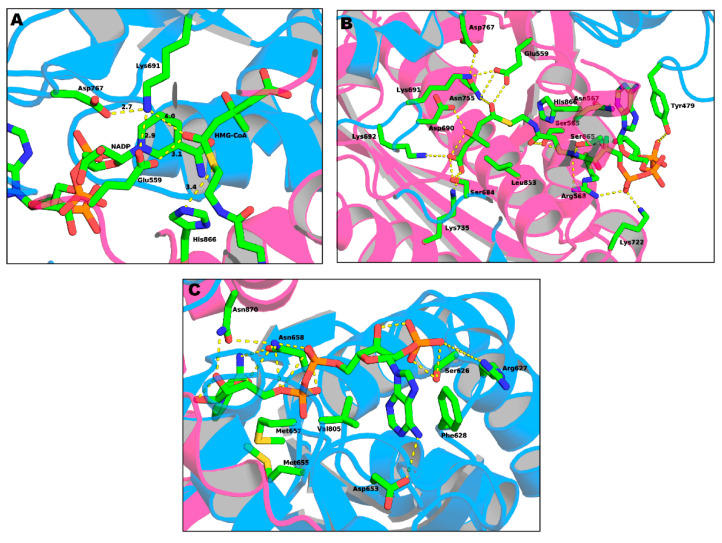
Representation of: (**A**) the active site of HMG-CoAR; (**B**) the binding sites of the HMG portion of HMG-CoA; and (**C**) the binding sites of NADP (PDB entry 1DQ9).

The binding site of CoA (Figure 10B) is positioned in the L-domain of one monomer. The ADP moiety of CoA binds near the surface of the enzyme, in a pocket lined with positively charged residues. The residues that are involved in the CoA binding pocket are Ser565, Asn567, Arg568, Lys722, Ser865, His866 and Tyr479 (this last residue comes from the neighboring monomer). The side chain of Tyr479 is of particular importance, as it interacts with the adenine base of CoA through π–π stacking while its hydroxyl group makes a hydrogen bond with the 3’-phosphate of the ribose moiety [49].

The NADPH (Figure 10C) binds to the S-domain of the opposing subunit in which the HMG-CoA binding pocket is located. Residues Ser626, Arg627, Phe628, Asp653, Met655, Gly656, Met657, Asn658 and Val805 come from the S-domain and residues Asn870 and Arg871 from the neighboring monomer. In the presence of NADPH, a conformational change in the C-terminus of the enzyme is observed which leads to the closure of the active site [49].

**Figure 11:**
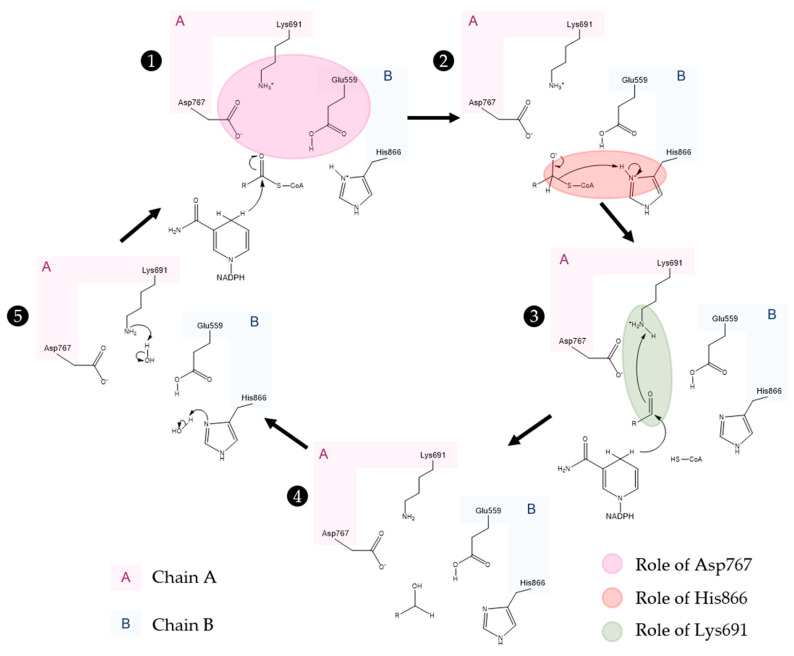
Currently accepted catalytic mechanism of HMG-CoAR [56]. The catalytic residues are Lys691, Glu559, Asp767 and His866; chain A is highlighted in pink and chain B is highlighted in blue. Glu559 and His866 are restored in a following step by deprotonation of adjacent water molecules.

Lastly, the HMG binding site (Figure 10A) is located at the interface of the two monomers. This binding pocket is formed by residues Ser684, Asp690, Lys691, Lys692 and Asp767 from one subunit and Glu559, Lys735, Asn755, Leu853 and His866 from the other. This is also the catalytic site of the enzyme. The currently accepted mechanism of HMG-CoA is present in Figure 11.

As observed in the mechanism shown in Figure 11, the enzyme HMG-CoAR enables the reduction of thioesterified HMG-CoA to mevalonate, using two molecules of NADPH for successive hydride transfers. The first step results in the formation of mevaldyl-CoA hemi-thioacetal, followed by the formation of the mevaldehyde, which occurs with the collapse of the thiohemiacetal and formation of CoA-SH (protonation of the thiol anion by Hys866). In the final step, the second NADPH reduces mevaldehyde to mevalonate [20,27,33,48,49,56].

The perception of this complex mechanism has changed through the years. In an earlier work, Tabernero et al. proposed a similar mechanism to the one depicted in Figure 11 for the *P. mevalonii* enzyme, in which Lys267 was proposed to be the general acid that stabilizes the mevaldyl-CoA intermediate [48]. As shown in Figure 6 and their work, it is not obvious to find the correct association between this residue from *P. mevalonii* and other HMG-CoARs using sequence homology. This was also observed by Haines et al., as their alignment is different from ours and the previous work [27]. In this latter work, they observed that the reduction of mevaldyl-CoA was performed with the aid of Glu83 (which is equivalent to Glu559 in the human enzyme), instead of Lys267. This work is supported by the position of the active site human residues as: (i) negatively charged intermediates can be stabilized by the positive charge of Lys691; (ii) the proximity between one of the side chain oxygens of Glu559 and the carbonyl oxygen of HMG suggests that this residue is protonated; (iii) the negatively charged Asp767 plays a critical role, as it is close enough to the glutamate to influence its pKa value, as well as being able to stabilize through ionic interactions the Lys691 side chain in the active; and (iv) the His866 can make a hydrogen bond with CoA thiol [27]. The mechanism proposed in Figure 11 follows the line of an QM/MM study, in which several approaches were tested. Oliveira et al. concluded that the correct mechanism is similar to the one proposed by Tabernero, however some structural features of Haines were used [56]. The presence of a neutral Glu559 in the active site allows for this residue to be more distant from the positively charged His866, as Glu559 and Asp767 form a hydrogen bond. Thus, Glu599 is no longer in an appropriate position to stabilize the mevaldyl-CoA intermediate, as previously suggested [27]. From the models tested by Oliveira et al., the one with the neutral Glu599 presented the lowest activation free energy for the reaction mechanism in Figure 11, in which the protonation of mevaldyl-CoA is performed by Lys691 [56].

Considering the structural differences between both classes of HMG-CoAR, and, consequently the different positions of the catalytic residues, it is safe to assume that the reaction mechanisms will be different. *P. mevalonii* enzyme is responsible for the reverse reaction of the human variant, and it has been shown that it is possible for this prokaryote to grow on mevalonate only [57]. The *cis*-loop found in Class I reductases, which is key for the positioning of the catalytic residues, is missing in this version of the protein. Instead, the position of His381 (analogous to His866) is approximated to the active site by the closure of the flap domain, i.e., the C-terminal 50 residues of Class II. This motion also alters the binding of the substrate and the cofactor [49,58].

## 4. Regulation of HMG-CoAR

HMG-CoAR is one of the most regulated enzymes in our body. The regulation can be achieved in four different ways: transcription of the enzyme’s gene [59,60,61,62,63,64]; translation of its mRNA [65]; degradation of the functional enzyme [66,67,68,69,70,71,72,73]; and modulation of its activity [74,75,76].

The transcription of HMG-CoAR gene and the rate of synthesis of this enzyme is controlled by the sterol regulatory element binding proteins (SREBPs). These proteins are commonly anchored in the ER membrane in a complex that is coupled with another two proteins, the SREBP cleavage activating protein (SCAP) and insulin induced gene protein (INSIG). The latter two proteins act as sterol sensors and render SREBPs inactive when they are bound together [3,50,61]. The complexed form occurs when a high concentration of cholesterol (or other sterols) is present in the cell. SCAP binds to cholesterol, which leads to a structural change that is stabilized by the INSIG proteins present in the ER. Upon the decrease of the cellular concentration of sterols, INSIG unbinds SCAP, which in turn escorts SREBP, with the aid of secretory proteins, to the Golgi complex [51,62,63,64].

Inside the Golgi complex, SREBP is sliced in two positions by specific proteolytic cleavages. This cleavage releases the N-terminal basic helix-loop-helix domain, which is now free to enter the nucleus behaving as a transcriptional factor and is able to recognize certain sequences of DNA called sterol-regulatory elements (SRE). When this transcription factor binds to them, it promotes the transcription of the HMG-CoAR gene, as well as other proteins used for lipid synthesis [63,64,65].

In addition to the transcriptional regulation, HMG-CoAR is also regulated via post-translational mechanisms. Following the increase in cellular sterol content, the degradation of HMG-CoAR is activated. INSIG once again has a key role in this process. When the levels of sterol are high enough, both SCAP and HMG-CoAR compete to bind INSIG. When SCAP binds to SREBP, the proteolytic release is shut down, and, when HMG-CoAR binds to INSIG, Lys248 of the human HMG-CoAR is ubiquitinated and the protein is then quickly degraded through a ubiquitin-proteasome mechanism [66,67,75]. More recently, it has been suggested that C4-dimethylated sterol intermediates produced during the mevalonate pathway can suppress SREBP cleavage and promote HMG-CoAR degradation, whereas cholesterol is a relatively weak inducer [77,78].

The catalytic activity of HMG-CoAR can also be modulated by phosphorylation [79,80]. Next to His866, one of the active site residues, there is a Ser872, which can be phosphorylated. The phosphorylation of this residue leads to the decrease of the catalytic activity of HMG-CoAR, since it decreases the affinity of the enzyme to NADPH. The position of the serine, so close to the catalytic histidine, is well conserved in superior eukaryotes, which suggests that the phosphoserine can interfere with the ability of histidine to protonate coenzyme A thioanion before it is released from the active site [75,76]. Alternatively, it is supposed that the phosphoserine can also prevent the closure of a C-terminal region, which is responsible for facilitating catalysis. The subsequent dephosphorylation of this serine completely restores the catalytic activity of HMG-CoAR [38,49].

## 5. HMG-CoAR Inhibitors

To try to diminish the escalating number of deaths caused directly or indirectly by the high levels of blood cholesterol, researchers started to investigate the best way to help reduce these levels. In fact, simply controlling the ingestion of cholesterol containing food was not enough to control its concentration in the blood [47,81]. Initial treatments to hyperchloremia used several approaches, such as bile-acids sequestrants, nicotinic acid, fibrates and probucol. The search for better cholesterol lowering drugs was maintained, due to the relative low efficiency of these medications.

### 5.1. Statins: The Most Common Inhibitor

In the mid 1970s, compactin, the first HMG-CoAR inhibitor, was discovered by Endo and coworkers, and the drug was tested for its efficiency as a cholesterol lowering medicine [82,83,84]. As the results were favorable, development of new and improved statins rose exponentially and soon after statins became the most used drug to control high levels of blood cholesterol, thereby reducing heart attacks and prolonging life in humans with atherosclerosis [85,86,87].

**Figure 12:**
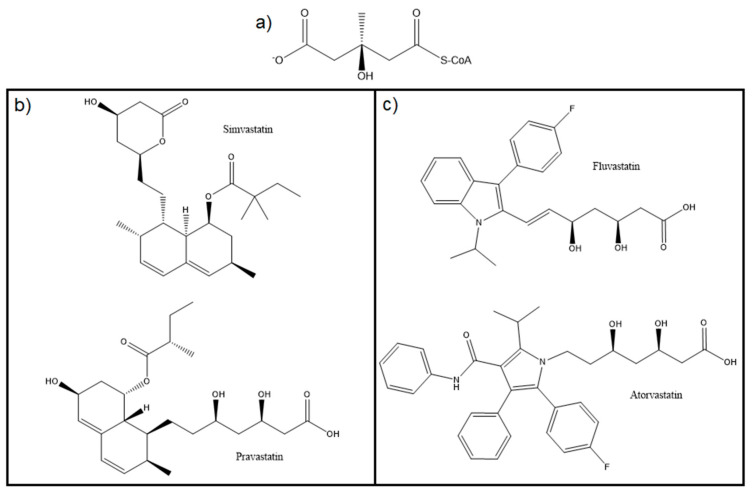
Structures of: (**a**) HMG-CoA; (**b**) Type I statins; and (**c**) Type II statins.

Statins are potent competitive inhibitors of HMG-CoAR and can be divided in two types, according to their origin [49,88,89]. Type I statins (Figure 12b), e.g., lovastatin, pravastatin and simvastatin, are natural fungal products and Type II statins (Figure 12c) are fully synthetic. All statins have similar structures to HMG (Figure 12a) and are covalently linked to a rigid hydrophobic group. When administered, the HMG-like moiety of these drugs is in an inactive form, which is later hydrolyzed in-vivo by cellular enzymes, i.e., esterases [90]. Type II statins are characterized by the presence of larger hydrophobic regions and attached fluorophenyl groups, as it is possible to observe in Figure 12c [91].

**Figure 13:**
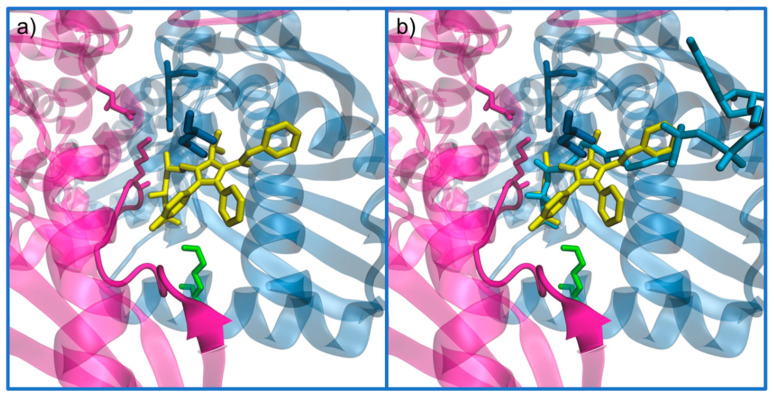
Demonstration of the active site residues when a statin (atorvastatin) is bounded (PDB code 1HWK): (**a**) only atorvastatin is presented in yellow; and (**b**) HMG-CoA is also bound to more easily compare the inhibitor with the natural substrate (PDB entry 1HWK and 1DQ9). These structures follow the representation and color code of the dimer in Figure 6, as chain A and B are colored in magenta and blue (transparent to better see details), respectively.

The ability of statins to inhibit HMG-CoAR arises from their similarity with the substrate, which leads to a competition towards the HMG binding site of the enzyme between HMG-CoA and statins. Even though the hydrophobic part of statins differs greatly from CoA portion of the substrate, it also blocks the access of HMG-CoA to the binding site (Figure 13). Thus, the affinity of this enzyme for statins is slightly higher than its affinity for the substrate [92].

The usage of statins may lead to some beneficial effects to the patients, such as plaque stabilization and anti-inflammatory and antithrombotic effects, among others [93]. These pleiotropic effects have and continue to be studied to repurpose statins in the treatment of other diseases, such as cancer [94] and regeneration of bone defects [95]. Nevertheless, there are several adverse side effects linked to statins [96], including skeletal muscle-related toxicity, cataracts, vascular lesions in the central nervous system and testicular degeneration and new-onset type 2 diabetes mellitus [97,98,99], or even lethal ones, e.g., rhabdomyolysis. Fatalities due to this disease led to the withdrawal of cerivastatin from the market in 2001 [100]. The mechanisms through which these adverse effects develop is still not quite understood and remains a topic of debate to this day. It is suggested that many of these side effects are due to the depletion of mevalonate-derived intermediates, which is a direct consequence of the inhibition of HMG-CoAR and the interruption of the mevalonate pathway. Despite all the possible side effects, this did not preclude atorvastatin from being the one of the most profitable and prescribed drugs in the world this millennium, which demonstrates that high blood concentration of cholesterol is a very serious problem that humankind is facing today [101,102].

The research on the side effects derived from the prescription of statins has been one of the major objectives of researchers in recent years, and it has been demonstrated that these drugs are also beneficial in fighting diseases other than hypercholesterolemia. For example, the inhibition of HMG-CoAR and subsequent depletion of melavonate-derived isoprene metabolites, which are essential for cell proliferation in both normal and tumor cells, has led to the conclusion that statins can be used as anticarcinogens [94,103,104,105,106,107,108]. In Alzheimer’s disease, the presence of hypertension, elevated plasma total cholesterol, low-density lipoprotein cholesterol levels (LDL) and atherosclerosis accelerate the progression and aggravate the symptoms. This means that, even though statins are usually prescribed to patients with high blood levels of cholesterol, they can also be a useful tool in the fight against other prevalent diseases [109,110].

### 5.2. Alternative Approaches

Statins have been used as the main inhibitors of HMG-CoAR for more than 30 years, despite the concerns that surround the adverse side effects that they can induce. However, there is still a need for further improvements in the treatment of hypercholesterolemia, which may or may not involve the usage of statins.

Several new methodologies have been reported for lowering blood cholesterol, which are focused on the inhibition of the synthesis of cholesterol [111,112,113], reducing its absorption [114,115,116], inducing reductase degradation [69,70,71,72,73] and limiting the synthesis of LDL [117,118,119]. SR-12813 and apomine (SR-45023A) are synthetic compounds that have been demonstrated to be powerful tools in this particular task by inducing HMG-CoAR degradation [69,70,72]. Another alternative is the chemical breakdown of HMG-CoAR using small-molecules proteolysis through the use chimeras (PROTACs) with subsequent inhibition in cholesterol production [73].

These approaches seem promising, and in the future one of them may become the preferred treatment, but currently the interruption of the mevalonate pathway through the inhibition of HMG-CoAR continues to be the most favored method.

Similar to statins, auranofin (AuRF), an anticancer agent, has been shown to inhibit HMG-CoAR, with half maximal inhibitory concentration at micromolar levels [120]. Peptide drugs, which are currently gaining more visibility in several therapeutic areas, have also shown some promising results as inhibitors of this enzyme, particularly the tetrapeptide PMAS, which has been shown to effectively inhibit HMG-CoAR (IC_50_ = 68 μM) [121]. It has also been demonstrated that meroterpenoids, ganoleucoins and triterpenes, compounds obtained from the medicinal mushroom *Ganoderma leucocontextum*, can have some activity towards the inhibition of this enzyme [122,123]. Infusions from *Vernonia condensata* Baker, and bay leaf (*Syzygium polianthum*) extracts, which were known to have the ability to reduce cholesterol levels, have also been a matter of study in recent years. In both cases, it was found that the preparations obtained from these plants contained compounds capable of binding HMG-CoAR and reducing its activity. In the case of *Vernonia condensata* Baker infusions, it was found this activity was due to caffeoylquinic acids [124], whereas, in the case of bay leaves, it was due to the presence of the phenolic compounds present in the extracts [125]. While it is true that most of these molecules show IC_50_ values much higher than that of common statins (in the micromolar range, as opposed to nanomolar for the statins), these can be a starting point for the study and introduction of a new class of statins.

Another interesting way to inhibit HMG-CoAR is by precluding the dimerization process. As discussed in Section 3, the enzyme consists of a “dimer of dimers”, in which each dimer contains two active sites. Therefore, preventing the formation of the different dimers avoids the formation of the active sites and, consequently, the formation of an active enzyme. To date, no dimerization inhibitors of HMG-CoAR are known, however there was a recent study that highlighted new druggable binding pockets that can be used for this purpose [126].

## 6. Conclusions

Cholesterol plays an essential role in cellular growth, membrane synthesis and differentiation. However, in the 1950s and 1960s, it became apparent that elevated concentrations of plasma cholesterol were a major risk factor for the development of coronary heart disease, which led to the early development of drugs that could reduce plasma cholesterol. One possibility was to reduce cholesterol biosynthesis, by inhibiting the rate-limiting enzyme in the cholesterol biosynthetic pathway, HMG-CoAR.

Currently, there is an impressive portfolio of studies regarding HMG-CoAR, and the details regarding the structure and catalytic mechanism of the enzyme are nowadays better understood.

Statins are the most popular HMG-CoAR inhibitors, particularly the fungal derivatives lovastatin, simvastatin and pravastatin and the synthetic fluvastatin and atorvastatin. These compounds are in the majority of cases well tolerated and have a finite and relatively safe side effect profile. Within the next few years, it is expected that statins will continue to be the main prescribed drug to treat hypercholesterolemia. However, the combination of statins with certain drugs can increase the risk of hepatotoxicity and myotoxicity effects, turning their use in some patients not favorable. Taking this into account, the development and application of new methods to inhibit HMG-CoAR is likely to play an increasingly important role in the treatment of hypercholesterolemia.

## Figures and Tables

**Table 1:** Currently known HMGCR crystal structures available in the Protein Data Bank, organized chronologically, starting with the most recently available.

PDB	Organism	Year	Resolution (Å)	Chain Length	Ligand(s)	Ref.
6HR8	*Methanothermococcus thermolithotrophicus*	2019	2.9	427	NADPH, PEG	[39]
6HR7			2.4	427	P6G, DTT	
6EEV	*Delftia acidovorans*	2018	1.5	429	MEV	[40]
6EEU			1.9	429	-	
6DIO			2.1	429	NAD	
5WPK	*Streptococcus pneumoniae*	2018	2.3	426	PE4, HMG	[41]
5WPJ			2.0	426	NADPH	
4I6Y	*Pseudomonas mevalonii*	2013	1.5	428	MEV	[21]
4I6W			1.7	428	1CO	
4I6A			1.9	428	HMG	
4I64			1.8	428	-	
4I56			1.5	428	1CZ	
4I4B			1.7	428	NAD, 1CO, 1CV	
3QAU	*Escherichia coli*	2011	2.3	458	-	n.a.
3QAE			2.3	458	-	
3CDB	*Homo sapiens*	2008	2.3	441	9HI	[42]
3CDA			2.1	441	8HI	
3CD7			2.1	441	882	
3CD5			2.4	441	7HI	
3CD0			2.4	441	6HI	
3CCZ	*Homo sapiens*		1.7	441	5HI	[42]
3CCW			2.1	441	4HI	
3CCT			2.1	441	3HI	
2R4F	*Homo sapiens*	2008	1.7	441	RIE	[43]
3BGL	*Homo sapiens*	2008	2.2	441	RID	[44]
2Q6C	*Homo sapiens*	2007	2.0	441	HR1	[45]
2Q6B			2.0	441	HR2	
2Q1L			2.1	441	882	
1T02	*Pseudomonas mevalonii*	2003	2.6	428	Lovastatin	[46]
1R7I	*Pseudomonas mevalonii*	2003	2.2	428	-	n.a.
1R31			2.1	428	MEV, CoA	
1HWL	*Homo sapiens*	2001	2.1	467	ADP, Rosuvastatin	[47]
1HWK			2.2	467	ADP, Atorvastatin	
1HWJ			2.3	467	ADP, Cerivastatin	
1HWI			2.3	467	ADP, Fluvastatin	
1HW9			2.3	467	ADP, Simvastatin	
1HW8			2.1	467	ADP, Compactin	
1DQA	*Homo sapiens*	2000	2.0	467	MAH, CoA, NADP	[38]
1DQ9	*Homo sapiens*		2.8	467	HMG	[38]
1DQ8			2.1	467	CoA, DTT, MAH	
1QAY	*Pseudomonas mevalonii*	1999	2.8	428	MEV, NAD	[48]
1QAX			2.8	428	HMG, NAD	
